# Pregnancy in Women With Preexisting Glomerular Diseases: A Single-Center Experience

**DOI:** 10.3389/fmed.2022.801144

**Published:** 2022-02-14

**Authors:** Smaragdi Marinaki, Stathis Tsiakas, Chrysanthi Skalioti, Eleni Kapsia, Sophia Lionaki, Kalliopi Vallianou, John Boletis

**Affiliations:** ^1^Department of Nephrology and Renal Transplantation, Medical School, National and Kapodistrian University of Athens, Laiko Hospital, Athens, Greece; ^2^Second Department of Internal Medicine, Faculty of Medicine, National and Kapodistrian University of Athens, Attikon Hospital, Athens, Greece

**Keywords:** pregnancy, glomerular diseases, lupus nephritis, outcomes, maternal, fetal, relapse

## Abstract

**Aim:**

Women with glomerular diseases are often of childbearing age. Besides lupus nephritis, data regarding pregnancy in patients with glomerular diseases are limited, posing a challenging task to attending nephrologists. This study aimed to investigate the pregnancy outcomes and the impact on the underlying glomerular disease among women followed in our institution.

**Methods:**

A single-center retrospective cohort study of women with biopsy-proven glomerular diseases who experienced pregnancy between 2010 and 2020. We analyzed data before, during, and after gestation.

**Results:**

A total of 22 women, 13 women with primary and 9 women with secondary glomerular diseases, were included in this study. Most patients (82%) had received immunosuppressive treatment at various times before pregnancy. All the women were in remission, either complete (62%) or partial (38%), with well-preserved renal function (82%) before conception. A total of 30 live births and 1 stillbirth were recorded; the rate of preterm delivery was 23%. Renal function and proteinuria remained stable during pregnancy. Preeclampsia was observed in 6.7% of patients and disease relapse in 6.9% of the pregnancies.

**Conclusion:**

Pregnancy was associated with a low frequency of adverse events in women with underlying glomerular diseases, provided they have quiescent disease and preserved renal function.

## Introduction

In women with underlying kidney disease, both the effect of pregnancy on renal disease and the effect of renal disease on pregnancy may be deleterious. Women with glomerular diseases (GDs) often have proteinuria, impaired renal function, or hypertension, factors which are associated with an increased risk for adverse pregnancy outcomes (1, 2). Despite the fact that most women with underlying GD are of childbearing age, there is a paucity of data about pregnancy and GDs. The only GD with sufficient evidence published today is lupus nephritis and treatment strategies for other GD are extrapolated mostly from limited studies (3–5). In primary glomerulonephritis (GN), with the exception of immunoglobulin A nephropathy (IgAN) (6, 7), most studies report on small numbers of patients from the older cohorts with varying rates of complications; therefore, it is difficult to draw definite conclusions about outcomes (8). In general, better outcomes have been reported for women with quiescent disease and preserved renal function at conception (4), while data on long-term follow-up before and after pregnancy are sparse. A systematic review, including pregnancy management guidelines for women with primary GD, published in 2017, has helped our understanding about the interplay between pregnancy and GD, but study heterogeneity and missing of important parameters, again preclude definite conclusions about the impact of specific factors on outcomes (9).

In this single-center study, we report the outcomes of 29 pregnancies and 30 live births in 22 women with primary and secondary GN, who were treated before and monitored during and after pregnancy in our institution. All the women had long-lasting nephritis before conception. We provide detailed description about prior treatment and disease status at conception and data about pregnancy monitoring and treatment as well as renal outcomes after delivery.

## Materials and Methods

### Data Extraction

This is a retrospective single-center study. From an electronic database comprising a total of 522 patients with immune-mediated GDs in 2020, we identified 22 women with biopsy-proven primary or secondary GD who had at least one completed pregnancy. All the 29 pregnancies occurred after 2010 and were closely monitored and actively treated when necessary. All the patients had long-lasting GD before pregnancy onset and had been diagnosed, followed, and treated in our institution starting as early as in 1998. Data were collected for type of GD, GD therapy, and GD status from first diagnosis until conception. During pregnancy, an intensified, predefined visit schedule with extensive workup was introduced in all of them. Data about pregnancy management strategy and outcomes were analyzed. We were in close contact with all the treating obstetricians and data about fetal status during pregnancy, delivery, obstetrical complications, and fetal outcomes were obtained systematically and recorded in our charts. After delivery, women continued their follow-up in our center and renal outcomes until the end of 2019 were analyzed as well. All the women provided a written informed consent and this study was approved by the Local Ethics Committee.

The *aim of this study* is to highlight the impact of preconception treatment and counseling and optimized pregnancy management on outcomes in women with different subtypes of immune-mediated GDs.

### Outcomes

The primary study endpoints were fetal and maternal outcomes and the secondary endpoints were maternal outcomes regarding renal parameters after delivery.

*Adverse fetal outcomes* included fetal death; preterm delivery, defined as delivery before completion of the 37th gestation week and classified as late preterm (34–37 weeks), moderate preterm (32–34 weeks), very preterm (28–32 weeks), and extremely preterm (<28 weeks); low birth weight (<2.5 kg) and very low birth weight (<1.5 kg) (10); neonatal intensive care unit (NICU) admission; and neonatal lupus syndrome.

*Adverse maternal outcomes* included nephritis flares, pre-eclamptic syndromes, worsening of renal function and/or proteinuria during pregnancy, gestational diabetes and hypertension, and obstetrical complications.

*Renal parameters and status of the underlying GD* were also monitored until the end of follow-up in all the women.

Renal parameters were recorded at the onset of pregnancy (baseline), at peak during pregnancy, immediately postpartum, and at the end of follow-up and were assessed as follows: renal function by use of serum creatinine at all the time points and also by estimated glomerular filtration rate (eGFR) calculated by the Chronic Kidney Disease Epidemiology Collaboration (CKD-EPI) equation before and after but not during pregnancy. Urine protein was measured in 24-h urine collection and presence of active or inactive urine sediment was examined. GD status was assessed based on these parameters and complete or partial remission or disease flare was defined according to validated criteria for each one. In patients with primary GD, complete remission was defined as a reduction of proteinuria to <0.3 g/d and normal serum creatinine; partial remission was defined as a 50% decrease of proteinuria and urine protein levels 0.3–3.5 g/d and improved or stable serum creatinine. In patients with lupus nephritis, complete remission was defined as a reduction of proteinuria to <0.5 g/d and return of serum creatinine to previous baseline value; partial remission was defined as a 50% decrease of proteinuria and urine protein levels 0.3–3.5 g/d and improved or stable serum creatinine. In antineutrophil cytoplasmic antibody (ANCA)-associated systemic vasculitis (AASV), remission was defined as the absence of microscopic hematuria and a stable or improved proteinuria and eGFR (11).

### Preconception Counseling

As all the patients had long-lasting disease with regular follow-up in our institution, once they expressed the wish to conceive, they received a counseling visit performed by a nephrologist. After careful assessment of potential risks and after having discussed them in detail with the patient, the decision was in most cases supporting, in some cases postponing, and in rare cases rejecting the will for pregnancy according to the current status of patients. Patients were advised to defer pregnancy until the disease was well-controlled for at least a year [i.e., complete or partial remission of GD according to the Kidney Disease: Improving Global Outcomes (KDIGO) guidelines, no flares or relapses within the past year] or in cases of advanced chronic kidney disease (CKD) (eGFR < 45 ml/min/1.73 m^2^) (11). Almost all the decisions were taken in agreement of the physician and the patient, but in some cases the patient insisted and indeed, proceeded with pregnancy despite having been instructed to withhold.

### Treatment of Underlying Nephritis Before Pregnancy

Immunosuppressive medications, duration of immunosuppressive therapy, and time from discontinuation until conception or supportive treatment only with renin-angiotensin-aldosterone system (RAAS) blockade or angiotensin receptor blockers (ARBs) were recorded.

### Follow-Up During Pregnancy

A first visit was scheduled immediately after conception and at predefined time intervals after that: once a month until the 20th gestation week, every 15 days from the 20th to the 28th gestation weeks, and once a week from the 28th gestation week until delivery, respectively. Besides routine laboratory investigation, 24-h urine protein, office and home blood pressure measurement, weighting and clinical examination, and contact with the treating obstetrician were performed at every visit. Medical treatment during pregnancy was administered in cooperation with the obstetrician and also recorded in our charts at every visit.

In patients with lupus nephritis, extensive immunological workup including anti-dsDNA antibodies, serum complement factors C3 and C4, antiphospholipid antibodies (APAs) [anticardiolipin immunoglobulin G (IgG) and immunoglobulin M (IgM), anti-β2 glycoprotein I (β2GPI) IgG and IgM], and anti-Ro/Sjögren's syndrome-related antigen A (SSA) and anti-La/SSB antibodies was assessed at first pregnancy visit and by indication after that and systemic lupus erythematosus (SLE) disease activity index (SLEDAI) was calculated. Rheumatologic consultation was considered in case of severe extrarenal manifestations.

## Results

We identified 29 pregnancies in 22 women, all with preexisting, biopsy-proven primary and secondary GD. Demographic characteristics and kidney biopsy diagnoses are shown in Tables 1, 2. All the patients had long-lasting disease with median time from GD diagnosis until pregnancy of 103 (range 24–252) months.

**Table 1:** Demographic and baseline characteristics.

**Patients**	**Race**	**GD**	**Age at GD** **diagnosis**	**Age at** **pregnancy** onset^*^	**sCr at** **pregnancy** **onset**	**uPR at** **pregnancy** **onset**	**BMI at** **pregnancy** **onset**	**Comorbidities**
1	Caucasian	IMN	18	34 39	0.6 0.66	1.27 1.59	21 22	Hypertension
2	Caucasian	IMN	29	32 36	0.6 0.6	1.37 0.19	24 25	None
3	Caucasian	IMN	34	37	0.57	0.13	24	Hypertension
4	Caucasian	IMN	28	34	0.76	0.19	22	None
5	Caucasian	FSGS	22	32 34	0.6 0.6	0.12 0.05	26 29	None
6	Caucasian	FSGS	33	40	1.6	0.20	22	Hypertension
7	Caucasian	FSGS	14	30	1.3	0.89	32	Hypertension
8	Caucasian	FSGS	21	35	0.6	0.81	20	Hypertension
9	Caucasian	IGMN	18	30	0.6	0.91	23	None
10	Caucasian	MCD	31	33	0.6	0.16	22	None
11	Caucasian	MCD	29	39	0.59	0.04	24	None
12	Caucasian	IGAN	24	29	0.6	0.19	20	None
13	Caucasian	IGAN	16	36	2.0	1.06	20	None
14	Caucasian	LN	28	41	0.6	0.18	23	None
15	Caucasian	LN	19	32 33	0.6	0.8	20	None
16	Caucasian	LN	22	25	0.6	0.08	24	None
17	Caucasian	LN	29	34 36 38	0.6 0.7 0.6	0.10 0.05 0.16	23 23 22	None
18	Caucasian	LN	28	37	0.69	0.19	29	None
19	Caucasian	LN	14	34	1.31	0.65	20	None
20	Caucasian	LN	21	28	0.64	0.64	29	None
21	Caucasian	AAV	27	32 36	0.11 0.79	0.6 0.67	30 28	None
22	Caucasian	AAV	30	37	1.27	0.84	29	None

*IMN, idiopathic membranous nephropathy; FSGS, focal segmental glomerulosclerosis; IGMN, IgM nephropathy; MCD, minimal change disease; IGAN, IgA nephropathy; LN, lupus nephritis; AAV, antineutrophil cytoplasmic antibody-associated vasculitis; GD, glomerular disease; sCr, serum creatinine; uPR, urine protein; BMI, body mass index*.

* *The upper number of each cell corresponds to the age of the first pregnancy*.

**Table 2:** Diagnosis of glomerular disease (*n* = 22).

**Kidney biopsy diagnosis**	**No. of patients** **(*n* = 22)**	**Percent of patients (%)**	**Median time from GD diagnosis until pregnancy (months)**
Membranous nephropathy	4	18	69
Focal segmental glomerulosclerosis	4	18	114
IgM nephropathy	1	5	150
Minimal change disease	2	9	65
IgA nephropathy	2	9	153
Lupus nephritis	7	32	107
ANCA-associated vasculitis	2	9	85

### Previous Treatment of the Underlying GD

In the majority of patients, 18 out of 22 (82%) patients had received immunosuppressive therapy before the onset of pregnancy. In total, 8 patients had received cyclophosphamide (CYC), 3 patients had received calcineurin inhibitors (CNIs), 4 patients had received mycophenolic acid (MPA), 4 patients had received only steroids, 4 patients had received rituximab, and 2 patients had received azathioprine. Four patients, two patients with membranous nephropathy, one patient with focal segmental glomerulosclerosis (FSGS), and one patient with IgM nephropathy, all with subnephrotic proteinuria for long, had received only RAAS blockade.

### Disease Status at Presentation

At pregnancy onset, all the patients had remission of GD, either complete, 18 out of 29 (62%) or partial in the remaining 11 out of 29 (38%) pregnancies, respectively. None of the patients was on immunosuppression (IS) at conception. Median time from IS discontinuation until conception was 22 (0–168) months. RAAS inhibitors were withdrawn in all the patients as soon as they started planning to conceive or immediately upon notification in case of an unplanned pregnancy. GD status and therapy from diagnosis until pregnancy are given in Tables 3, 4.

**Table 3:** GD status and therapy from diagnosis until pregnancy. Patients with primary GD (*n* = 13).

**Disease**	**Diagnosis of GN (year)**	**Pregnancy onset (year)**	**Time since Diagnosis (months)**	**Therapy**	**Duration of immuno-suppression (months)**	**Time since immuno-suppression discontinuation (months)**	**Duration of remission prior to pregnancy (months)**	**Disease status at pregancy**
IMN	1997	2013 2018	192 252	RAAS inhibitors	-	-	5 65	PR
IMN	2011	2014 2017	29 65	RAAS inhibitors	-	-	24 60	CR
IMN	2016	2019	32	CYC+CS	6	22	18	CR
IMN	2012	2018	57	CYC+CS+ CSA+RTX	57	14	34	CR
FSGS	2000	2010 2012	114 138	CSA+CS	72	24 46	48 70	CR
FSGS	2006	2013	84	CSA+CS	60	13	48	PR
FSGS	1998	2014	184	CS	18	168	57	PR
FSGS	2003	2017	48	RAAS inhibitors	-	-	36	PR
IGMN	2003	2015	150	NONE	-	-	14	PR
MCD	2011	2013	24	CS	12	12	23	CR
MCD	2009	2018	106	CS	12	24	31	CR
IGAN	2008	2013	66	CYC+CS	16	48	60	CR
IGAN	1993	2013	240	CS	NA	6	6	PR

*IMN, idiopathic membranous nephropathy; FSGS, focal segmental glomerulosclerosis; IGMN, IgM nephropathy; MCD, minimal change disease; IGAN, IgA nephropathy; RAAS, renin angiotensin aldosterone system; CYC, cyclophosphamide; CS, corticosteroid; CSA, cyclosporine; RTX, rituximab; GD, glomerular disease*.

**Table 4:** GD status and therapy from diagnosis until pregnancy. Patients with secondary GD (*n* = 9).

**Disease**	**Diagnosis of GN (year)**	**Pregnancy onset (year)**	**Time since diagnosis (months)**	**Therapy**	**Duration of immuno-suppression (months)**	**Time since immuno-suppression Discontinuation (months)**	**Duration of remission prior to pregnancy (months)**	**Disease Status at Pregancy**
LN	1998	2011	156	CYC+MPA+ CS+AZA+ RTX	156	0.8	48	CR
LN	1999	2012 2013	156	MPA+CS	60	0.8 3	20 7	PR PR
LN	2012	2015	46	MPA+CS	45	0	37	CR
LN	2009	2014 2016 2018	62 91 109	MPA+CS +RTX	59	3 34 48	56 90 103	CR CR CR
LN	2007	2016	106	MPA+CS	88	16	93	CR
LN	1998	2018	243	PLEX+CYC+ MPA+CS+ AZA+RTX	192	46	60	PR
LN	2011	2018	80	CYC+MPA+CS	53	27	76	CR
AAV	2010	2015 2019	60 103	CYC+MPA+ CS	48	12 57	54 96	CR CR
AAV	2012	2019	85	CYC+MPA+CS	30	55	72	CR

*LN, lupus nephritis; AA, antineutrophil cytoplasmic antibody-associated vasculitis; CYC, cyclophosphamide; CS, corticosteroid; MPA, mycophenolic acid; AZA, azathioprine; RTX, rituximab; PLEX, plasma exchange; GD, glomerular disease*.

Ten pregnancies were recorded in 7 patients with SLE nephritis. At onset, 4 and 5 patients had only slightly elevated dsDNA titers and low complement (C3 and/or C4) levels, respectively. One patient was positive for anti-Ro/SSA Abs and one patient was positive for APAs. Median SLEDAI index was 2.0.

Median age of patients at conception was 34 years (range 25–41 years) with 10 out of 29 (35%) patients being over 35 years. Median body mass index (BMI) was 23.2 kg/m^2^ (range 19.9–32 kg/m^2^), 7 out of 29 (24%) patients were overweight and one patient was obese. Most women had a natural pregnancy, while 4 out of 22 women conceived after assisted reproductive technology (ART), which comprised ovulation induction (OI) and *in vitro* fertilization (IVF).

A total of 29 pregnancies were recorded in 22 women, resulting in 30 live births: two out of 29 were twins, both in women after ART, while 6 patients had more than one pregnancy; five had 2 and one even three consecutive pregnancies.

Pregnancy outcomes are shown in Tables 5, 6.

**Table 5:** Fetal/neonatal outcomes.

	**No**.	**Percentage**
**Fetal Loss**		
Neonatal death	none	0
Stillbirth at >20 weeks	1	3.4
**Preterm delivery**		
Late (34–37 weeks)	6	20
Moderate (32–34 weeks)	1	3.4
**Low birth weight**		
2.0–2.5 kg	5	16.6
1.5–2.0 kg	4	13.3
**ICU admissions**	6	20

*Percentage per total number of newborns (n = 30)*.

**Table 6:** Maternal outcomes.

	**No**.	**Percentage**
**Mode of delivery**		
Cesarean section	24	82
Vaginal delivery	5	18
Pre-eclamptic syndrome	2	6.7
Nephritis flare	2	7
LN	1	
FSGS	1	
Gestational diabetes	1	3.4
Increase in creatinine (>25% from baseline)	2	7
Gestational hypertension	1	3.4
Obstetric complications	3	10

*Percentage per total number of pregnancies (n = 29)*.

### Fetal Outcomes

A total of 29 pregnancies resulted in 30 live births. There was no neonatal death, while there was one stillbirth in the 23rd week due to maternal chorioamnionitis. Median gestation time was 37 weeks (range 23–39 weeks) with a preterm delivery rate of 21% (6 out of 30) for premature delivery before the 37th week and 3.4% (1 out of 30) for delivery before the 34th week, respectively.

Out of the 30 live newborns, 5 (16.6%) had low (<2.5 kg) and 4 (13.3%) had very low (<2 kg) birth weight. Of the 30 neonates, 6 (20%) were admitted to the neonatal intensive care unit (NICU).

Out of 10 pregnancies and 11 newborns in 7 patients with lupus nephritis, there was no case of neonatal lupus syndrome.

### Maternal Outcomes

There was no maternal death nor major complication as thrombosis, stroke, or sepsis in our cohort. Two pre-eclamptic syndromes occurred in 29 pregnancies (6.7%): one case of preeclampsia in week 36 in a patient with membranous nephropathy and one case of HELLP syndrome in week 32 in a patient with SLE nephritis. Both resulted in successful emergent delivery without major adverse outcome, neither for the neonates nor for the mothers. Two more patients flared during pregnancy: one with SLE nephritis in the 17th week of gestation and the second with FSGS immediately before delivery in week 37. Again, both the cases were treated successfully, with good outcomes for mother and child. One woman developed gestational diabetes and one woman developed gestational hypertension.

From the 29 deliveries, the majority, 24 out of 29 (83%) deliveries were with cesarean section (C-section), in some cases emergent, but in most cases elective, since they comprise the high-risk patient group and obstetricians consider the C-section the safer mode of delivery. Obstetrical complications occurred in three patients: one emergent C-section was performed in one woman in the 37th week due to acute placental hemorrhage, one more woman with twin pregnancy had placenta previa and also underwent emergent C-section, and the third developed an abdominal abscess after delivery with C-section. All the complications were treated successfully with good outcomes for both the women and the neonates.

### Renal Parameters

*Regarding renal function*, most patients in our cohort, 18 out of 22 (82%) patients had excellent renal function at baseline. Median creatinine in the entire cohort was 0.6 (range 0.5–2.0) mg/dl and eGFR (CKD-EPI) was 115 (range 30–120) ml/min/1.73 m^2^ at baseline. GFR was calculated with the CKD-EPI equation only at baseline and not during follow-up, since it is unreliable, especially late in the course of pregnancy. Renal function was preserved during and immediately after pregnancy as well as after 28 months of follow-up in all the 18 women.

**Figure 1:**
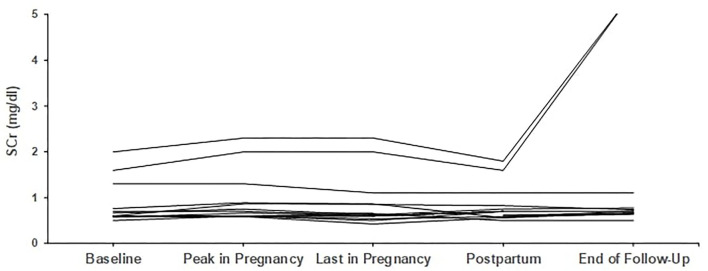
Trends in creatinine in patients with primary glomerular diseases (*n* = 13). Individual trends in serum creatinine levels at five time points: baseline, peak during pregnancy, last pregnancy visit, postpartum, and end follow-up.

**Figure 2:**
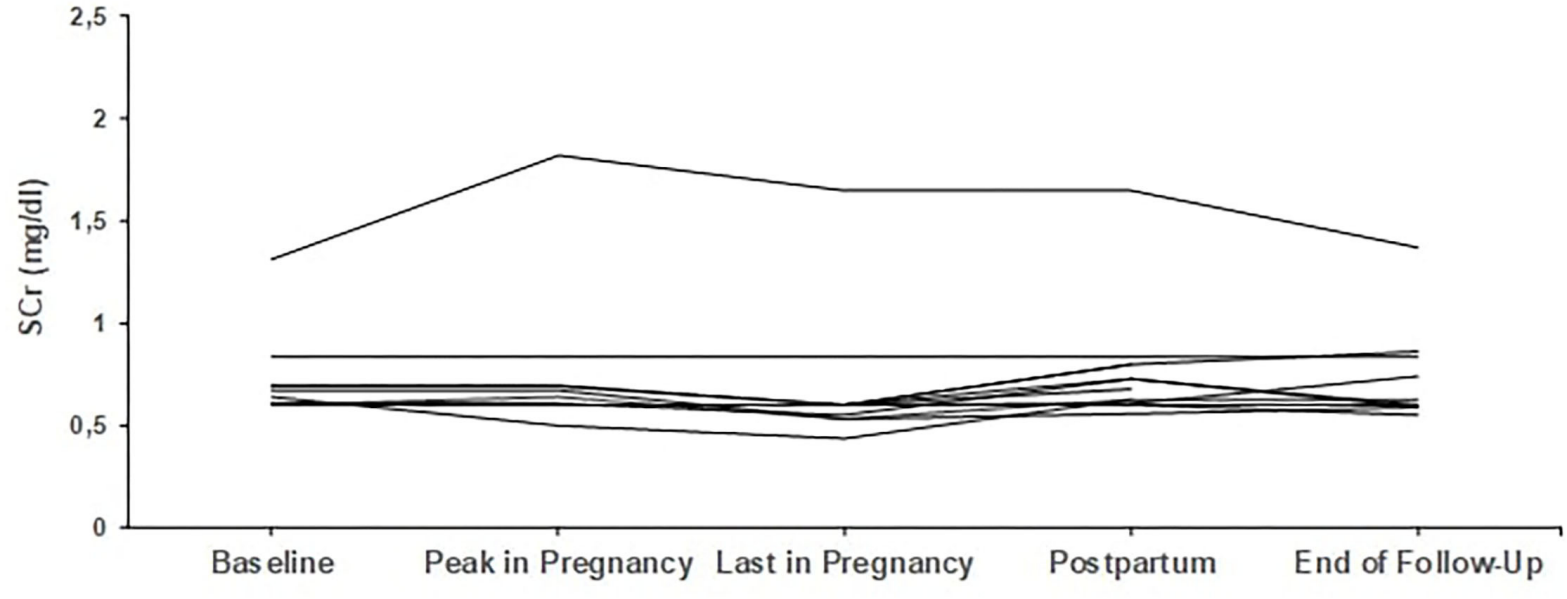
Trends in creatinine in patients with secondary glomerular diseases (*n* = 9). Individual trends in serum creatinine levels at five time points: baseline, peak during pregnancy, last pregnancy visit, postpartum, and end follow-up.

From the remaining four who started pregnancy with creatinine above 1.2 mg/dl, one patient started with 1.3 mg/dl and finished pregnancy with 1.1 mg/dl, while the other three patients deteriorated; more than 25% increase in creatinine was noticed in the first and second patient, with creatinine levels rising from 1.3 to 1.65 mg/dl and from 1.6 to 2.0 mg/dl, respectively, while the third patient had an increase from 2.0 to 2.3 mg/dl. The second and third patient, with the most impaired renal function at baseline, eventually progressed to end-stage renal disease (ESRD) 19 and 48 months after delivery. Trends in creatinine levels are given in Figures 1, 2.

**Figure 3:**
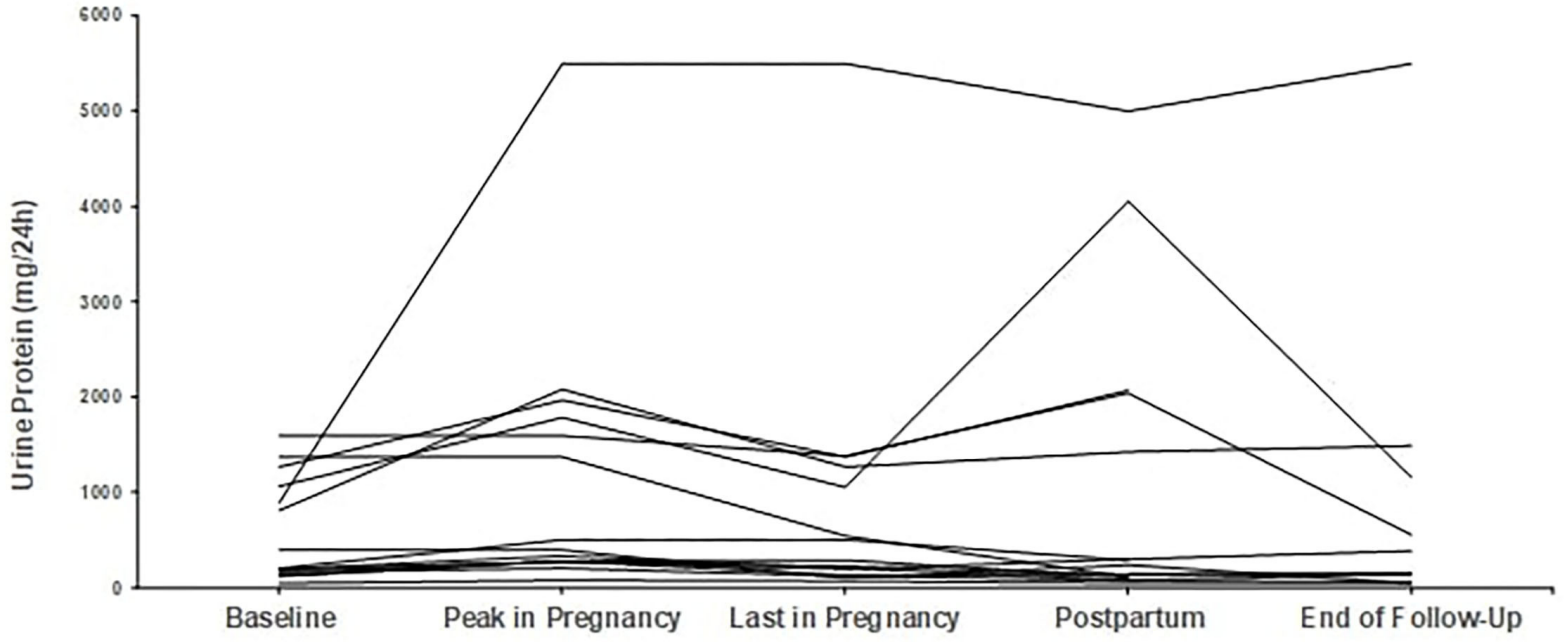
Trends in proteinuria in patients with primary glomerular diseases (*n* = 13). Individual trends in proteinuria at five time points: baseline, peak during pregnancy, last pregnancy visit, postpartum, and end follow-up.

**Figure 4:**
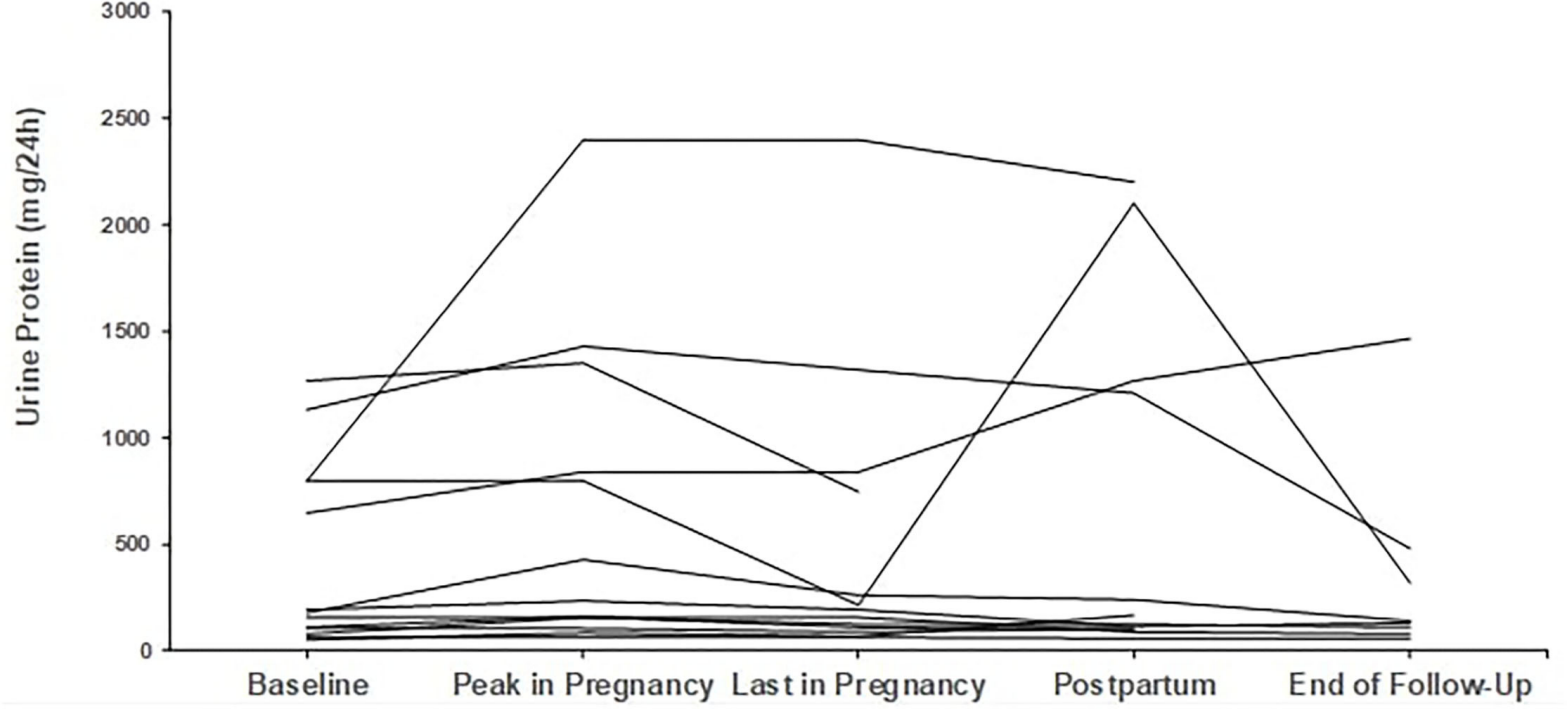
Trends in proteinuria in patients with secondary glomerular diseases (*n* = 9). Individual trends in proteinuria at five time points: baseline, peak during pregnancy, last pregnancy visit, postpartum, and end follow-up.

*Regarding proteinuria*, median baseline proteinuria was low, as expected since most patients were in remission. Median proteinuria was 193 mg/d (range 50–1,590 mg/d) at baseline and did not increase during pregnancy with a median value of 201 mg/d at the end. In 6 out of 29 pregnancies, proteinuria was slightly but not severely elevated, above 1 g/d (range 1.06–1.57 mg/24 h), at pregnancy onset; however, no further increase was noticed. Trends in proteinuria are given in Figures 3, 4.

## Discussion

In this single-center study of 29 pregnancies resulting in 30 live births in 22 women with preexisting GD, we could identify a low frequency of adverse maternal and fetal outcomes.

Regarding fetal outcomes, we had a live birth rate approaching 100%, with no neonatal death and only one stillbirth, due to chorioamnionitis, which cannot be attributed to the underlying GD. The rate of low birth weight in our cohort is 33% (7 out of 30 neonates). However, if we exclude the two twin pregnancies, both after ART, resulting in four newborns with very low (below 2 kg) birth weight, the rate is 16.6%, almost twice that of the general population, which for Greece has been reported at 9.3% in 2021 (12). As for twin pregnancies, rates of low birth weight and preterm delivery are 55 and 61% for pregnancies after ART and 50 and 57% in those who have conceived naturally (13). Median duration of pregnancy in our cohort was 37 weeks, with a rate of preterm delivery at 23.4%, again almost twice the rate of the general population of the country, which is at 12% in 2021 (14). From the 30 live newborns, 6 were admitted to the NICU (20%). Again, 4 out of the 6 were the two pairs of twins, who had been admitted due to very low birth weight. One more was admitted due to bradycardia and another due to low birth weight after delivery in the 32nd week because of preeclampsia of the mother. All were discharged without residual impairment.

Our rates were favorable considering prior reported clinical outcomes in women with underlying GD. There are not many studies assessing pregnancy outcomes in women with different subtypes of GD. Regarding former studies, in a review including 6 studies conducted in the late 1980's and comprising a total of 906 pregnancies in 558 women with primary GN, Jungers and Chauveau report a total fetal loss rate at 22% with perinatal (after the 26th week) death of 13% (15). This confirms the findings from smaller studies in the late 1980's to 1990's, which all report high rates of adverse fetal and maternal outcomes. Fetal loss rates are high, with live birth rates about 75% and rates of prematurity, intrauterine growth restriction (IUGR), and low birth weight are in the range of 25–60% (8, 16). Most worryingly, a recent study published in 2017, investigating 48 pregnancies in 43 women with different subtypes of GD, reports also high rates of adverse fetal and maternal outcomes: perinatal death occurred in 12.5%, prematurity rate was 48%, and IUGR at 12.5% (17).

As for maternal outcomes, we had also very good outcomes and few, only minor complications. We found a very low rate of preeclampsia (6.7%) as well as of nephritis flares (7%). Preeclampsia is one of the most common and feared complications of pregnancy in women with kidney disease. Reported preeclampsia rates vary broadly across studies, with the lower rates being about 7–8% and the highest reaching 25%, depending on diagnosis and disease status at conception (18–20). In the most recent study from North Carolina, the rate of preeclampsia was high, at 33% (17). Our pre-eclamptic syndromes were treated with emergent delivery in the 32nd and 36th gestational weeks with successful outcomes for both the mothers and newborns. At this point, it must be noticed that we were in close collaboration with the treating obstetricians and diagnosis of preeclampsia as well as decision about emergent delivery was taken after agreement of both the gynecologist and the nephrologist.

Two nephritis flares occurred during the 29 pregnancies: the first in the 37th gestational week in a patient with FSGS, manifesting with severe nephrotic syndrome, which was successfully handled with urgent delivery and the second in a patient with SLE nephritis, in the 17th gestational week, who developed subnephrotic proteinuria and active urine sediment. In the latter patient as well as in one more patient, with a diagnosis of AASV, who also developed subnephrotic proteinuria and active urine sediment in the 12th gestational week, we performed a renal biopsy. The main clinical indications for renal biopsy during pregnancy are unexpected deterioration of renal function, *de-novo* nephrotic syndrome, and suspicion of new-onset GD. Indications are reasonably strict, since an invasive procedure places both the woman and the embryo at risk. Our patients had subnephrotic proteinuria and no deterioration of renal function. However, both had reactivated prior inactive urine sediment and suffered from secondary GN, i.e., SLE and AASV, which are both conditions with a high relapse risk. Furthermore, the patient with SLE nephritis had discontinued IS only few days before the onset of pregnancy. In this patient, not surprisingly, kidney biopsy revealed an early nephritis flare with active, though mild-to-moderate class III lesions and she was started IS consisting of azathioprine and steroids. In the second patient with the AASV diagnosis, biopsy revealed only mild mesangial proliferation and a pauci-immune pattern on immunofluorescence; accordingly, no IS was given. Renal biopsy, especially early in the course of pregnancy, when a GD flare is more expected than a pre-eclamptic syndrome, may be extremely helpful for decision guidance in order not only to (re)initiate, but also to withhold otherwise unnecessary treatment. As for safety, both the biopsies were performed in the usual prone position without complications. Regarding complications, the only reliable study reports a low complication rate of 4.5%, comparable to that of the general population, in 111 pregnant women (21). Higher rates are reported late during pregnancy, especially after the 17th gestational week (22).

Renal parameters in means of renal function and proteinuria were excellent at baseline in the majority of patients and remained so until the end of pregnancy and still after 28 months of follow-up. From the three women with creatinine above 1.2 mg/dl at baseline, two women deteriorated and reached ESRD after 19 and 48 months, respectively, and the third women had an increase of more than 25% in creatinine, while delivery was before 10 months, so follow-up is still too short to draw conclusions about future outcome. Low proteinuria levels (median of 193 mg/24 h) were also sustained during pregnancy in the 6 women who had started pregnancy with levels above 1 g/24 h and even the effect of hyperfiltration-induced increase in proteinuria was not detected in our cohort. In the abovementioned study from North Carolina, O'Saughnessy and co-authors found a more than 50% increase in creatinine in 27% and doubling of urinary protein in 39% of pregnancies, respectively (17).

Very few studies have evaluated the impact of different GD subtypes on pregnancy outcomes with varying patterns (17, 23). In our cohort, numbers are too small to make such comparisons. However, to our point of view, in secondary GD, but especially in primary GD, main factors that determine successful pregnancy outcomes, more than diagnosis itself, are remission of either nephrotic or nephritic features and preserved renal function at onset.

There are several explanations for the good pregnancy outcomes in our cohort. It is not that our patients had mild disease. All had long-lasting nephritis with median duration from GN diagnosis until pregnancy of 8.5 years. From the 22 women, 7 women had SLE nephritis and 2 women had AASV, both the conditions associated with a long history, relapses, high immunosuppressive burden, and adverse pregnancy outcomes. Moreover, 82% of patients in this study had received IS for long (median of 58 months), 8 out of them with CYC.

The most critical point is that all the patients were in remission at conception. Quiescent disease is one of the most important features for successful pregnancy outcomes for mothers as for neonates. This is well-documented so far, primarily in SLE, but also in other secondary and primary GD (24–26).

Herein, we must underline the importance of preconception counseling. As already mentioned before, all the patients had long-term follow-up in our institution for preexisting GD. At the preconception visit, the final decision in means of proceeding or not with pregnancy was taken. Many patients had expressed the wish to conceive at different time points during their disease course and had postponed pregnancy if advised so, until it was considered safe. In accordance with this, it is the time interval between GD remission as well as the time interval between IS discontinuation and pregnancy onset; moreover, age of our patients at conception is older compared to that in most other series.

Another important issue for successful pregnancy outcomes in women with underlying GD is preserved renal function. The majority (82%) in our cohort had excellent baseline renal function, which was preserved not only during pregnancy, but also after 28 months of follow-up. Impaired renal function at pregnancy onset has been associated with higher rates of adverse pregnancy outcomes and accelerated maternal CKD. Conversely, in patients with preserved renal function, pregnancy does not negatively influence renal parameters and does not affect long-term renal function (8, 17). This has been shown in a multicenter Italian cohort of 223 pregnant women with primary diagnosis of IgAN, baseline creatinine below 1.2 mg/dl, and follow-up of 10 years (8). The reported outcomes in this cohort are very similar to ours, further supporting the evidence that disease remission and good renal function are major determinants of outcome.

Last but not least, patients in this study had a very close follow-up and there was continuous cooperation between treating physicians of a multidisciplinary team throughout pregnancy.

This study has several limitations: it is a retrospective study from a single institution, with a small number of patients and heterogeneity of the underlying GD. On the other hand, it is a very homogeneous cohort with long-term follow-up before and after pregnancy and close monitoring according to a predefined protocol for laboratory and clinical evaluation at scheduled visits during pregnancy.

## Conclusion

In women with underlying GDs, pregnancy is safe and should be encouraged, provided they have quiescent disease and preserved renal function.

## Data Availability Statement

The raw data supporting the conclusions of this article will be made available by the authors, without undue reservation.

## Ethics Statement

The studies involving human participants were reviewed and approved by Data Protection Manager (Laiko General Hospital of Athens, protocol code 118/31-3-2021). The patients/participants provided their written informed consent to participate in this study.

## Author Contributions

SM designed the study and drafted the article. ST, EK, and KV collected and analyzed the data. CS validated the data. SL and JB supervised the process and reviewed the final version of the article. All the authors provided contributions and approved the version of the article to be published.

## Conflict of Interest

The authors declare that the research was conducted in the absence of any commercial or financial relationships that could be construed as a potential conflict of interest.

## Publisher's Note

All claims expressed in this article are solely those of the authors and do not necessarily represent those of their affiliated organizations, or those of the publisher, the editors and the reviewers. Any product that may be evaluated in this article, or claim that may be made by its manufacturer, is not guaranteed or endorsed by the publisher.
